# Caries of the Human Teeth

**Published:** 1883-12

**Authors:** W. D. Miller

**Affiliations:** Berlin


### Independent Practitioner.

VOL. IV. DECEMBER, 1883. No. 12.

### Original Communications.

### CARIES OF THE HUMAN TEETH.

BY DR. W. D. MILLER, BERLIN.

READ BEFORE THE AMERICAN DENTAL SOCIETY OF EUROPE, AT COLOGNE.

In the beginning of this communication I wish to say what to most present may be self-evident ; that to any one who has made a moderately extensive study of the subject of dental caries, the phenomena associated with this lesion will be found to be so numerous as to render impossible any attempt to fully describe them all, much less account for their origin in the space of a few pages.

I shall therefore be compelled to pass rapidly over those results of my investigations which, having been already published, are perhaps more or less familiar to most of the gentlemen present, and dwell at greater length upon the results of more recent experiments, which have not yet been made public.

We may employ five different methods in studying this question.

*First.* The structure and chemical composition of the teeth, and the nature of the agents and reagents by which they are surrounded in the human mouth, are carefully considered, so that we may be able to form an intelligent conclusion as to the probable results produced by their action upon each other.

*Second.* Carious teeth, both in the fresh and dry state, are, in thick sections, subjected to a careful *macro*scopical examination.

*Third.* The anatomical changes produced by caries in the structure of the tooth are studied on *micro*scopical sections of the various tissues of the tooth.

*Fourth.* The exact chemical changes which have taken place in the diseased tissues are determined by proper chemical analyses.

*Fifth.* Pieces of sound tooth tissue are subjected to conditions as similar as possible to those found in the human mouth, for the purpose of artificially producing the phenomena of caries.

No one of these methods, taken alone, could constitute more than an imperfect treatment of the subject, but all taken together, especially if their evidence points in one direction, appear to furnish as complete a solution as we at present are capable of obtaining.

These different methods have all received consideration in my investigations, and shall be allowed their proper value in this exposition, though not in the order mentioned above.

At present, three different theories—the inflammatory, chemical, and parasitical—are being advocated with about equal zeal, to account for the phenomena of dental caries. To these may be added my own views, which are not in complete accordance with either of the above mentioned hypotheses.

Up to within a few months the conviction had become pretty general that the first stage of dental caries (if not the whole process) consisted in a decalcification of the tissue of the tooth by various acids. Later, however, a reaction arose in America, the object of which was to replace acids by an agent of a parasitical nature, but no attempt was made to prove that this agent had in reality the power of producing the effects which had previously been attributed to acids. On the other hand, the advocates of the parasitical theory of decay distinctly affirm that no acid is necessary, and that no decalcification takes place in the process of caries. This is the fundamental idea, and at the same time the fundamental error of the germ theory.

This reaction seems to have had its origin in certain tests of the saliva in cases where caries was rapidly advancing, where no acid condition was found, an experiment which is without significance, simply because the experimenter failed to state under what conditions and at what time the experiment was made, and because the absence of an acid condition of the saliva itself tells us nothing whatever as to what is going on between the teeth, or in fissures or cavities of decay. If any one should tell me that the saliva of a certain person whose teeth were decaying rapidly was not acid, I would readily accept the statement as a fact, but I do not see how I could found a theory of dental caries upon it. I will, moreover, show farther on, that a neutral, or but slightly acid reaction of the saliva where caries is rapidly advancing, and an acid reaction where little caries is present, are by no means necessarily at variance with an acid theory of decay.

We may put it down as an axiom, that whenever a mixture of saliva with saccharine or amylaceous substances, or a solution of either in normal saliva, *i. e.* in saliva as it is found in the human mouth, stands for from four to five hours at the temperature of the human body, as sure as once one is one an acid will be generated, and this acid will with equal surety have the power of decalcifying tooth tissue.

If a patient comes to us directly after eating, there is not so liable to be an acid reaction where food has lodged, as some hours afterward. If he has very carefully cleaned his teeth before coming, there is still less liability to an acid condition. If he goes to bed without cleaning his teeth, there will invariably be a strong acid reaction in the morning where food has remained about the teeth.

I have of late examined two hundred and thirty cavities of decay; two hundred and twenty-five were acid, four neutral and one alkaline. Meat in saliva becomes acid in four to five hours, neutral in ten to twelve, and alkaline in fifteen. This circumstance may account for an occasional temporary alkaline reaction.

The test for acid should be made in the following manner: Absorb the excess of moisture in the cavity, remove the outermost portions of the rests of food, and apply the litmus paper to the deeper parts which come less in contact with liquids in the mouth, or directly to the carious dentine itself ; the degree of acidity is often a source of great surprise.

I broke up several teeth, perfectly free from caries, but of different density, into fragments of various sizes, and put the pieces in a mixture of saliva and bread. The mixture was kept at a temperature of thirty-seven degrees Centigrade for three months, being renewed in that period five times. I then showed a number of these pieces to a well-known practitioner of thirty-three years standing, and asked him if they were not strange cases of caries. He replied, No ! he met with such cases daily. The dentine was, in many of the pieces, softened through and through, and in all to a considerable depth. Where the softening had penetrated through the dentine to the inner surface of the enamel, the latter was coated with a layer of white powder, exactly as we find it in natural caries; the edges of the enamel (where the pieces were fractured) were reduced to a powder from one-quarter to one-half mm. in thickness ; cracks in the enamel presented an opaque, whitish appearance, and in some cases could be penetrated by a fine-pointed instrument. At the neck of the tooth the dentine had become softened, though not as extensively as in the crown ; the edge of the enamel was rough and fragile, and in some cases an instrument could easily be inserted between it and the dentine ; on the grinding surface, where the tooth was of very poor structure and full of fissures and pits, the tissue had been reduced to a soft, cheesy mass, such as we so often meet with in third molars.

In two cases, evidently owing to a defect in the structure, the teeth were attacked on the cusps and reduced to a powder; where the acid had penetrated through the enamel, its effect was seen to spread out in all directions in the dentine ; where the enamel was very hard and dense, without crack or blemish of any kind, it had not even lost its lustre. In fact, all the phenomena of white decay were here reproduced with the greatest accuracy. If the mixture was allowed to stand until the reaction became alkaline, or if the pieces were exposed to the action of the air, or to various articles of food, such as coffee, tea, tobacco, juices of fruits, etc., all possible shades of color ever exhibited in carious dentine were produced.

This experiment shows clearly enough what a vast difference the structure, (density) of the tooth makes in its resistance to decalcification, and offers an explanation of the question why all teeth do not decay alike. A dense tooth, covered with enamel, intact at every point, would probably resist for years the action of an acid saliva, to which a soft, defective tooth would succumb in a few weeks.

It also shows that the process of decalcification may go on in cracks and fissures too fine for food to penetrate, in fact anywhere, where saliva holding starch or sugar in solution may find access, and be retained even by capillary attraction, there to undergo fermentation.

It might, therefore, happen that in a mouth containing soft, porous teeth, which were rapidly decaying, no strong acid reaction would be perceptible, because the acid soon after being generated would, for a great part at least, enter into combination with the lime of the tooth, whereas if the teeth were dense and protected by perfect enamel the acid would combine very slowly, or not at all, and the litmus might show free acid where there was no caries. This is, in theory at least, by no means an impossible case. On the contrary, it lies quite within the bounds of probability, and shows how careful we must be in drawing conclusions about the nature of caries, simply because now and then we, at the time of making the test, fail to discover as much acid as we think we ought to.

We should first answer the question : Has not the acid, or a great part of it, already spent itself upon some vulnerable part of the tooth ? The fact established by the above experiment, that the rapidity with which tooth tissue is acted upon by the acid of tooth caries is inversely proportional to some multiple of the density of the tooth, I consider to be of the greatest importance, as furnishing a key to the solution of the question of the great difference in the liability of the teeth in different persons, or of different teeth of the same person, to decay.

If we drop a lump of rock salt and one of ordinary table salt of the same size into a pail of water, the latter will disappear almost instantly; the former not until after the lapse of a comparatively long time. The difference, however, is probably no greater than would be seen in the action of the same acid upon a very dense and a very porous tooth. This difference is chiefly due to the much greater extent of surface exposed by the porous tooth; also to the fact that the saliva holding starch or sugar in solution may penetrate it and become acid by fermentation; and further, to the fact that a porous tooth containing a less amount of lime salts than a dense one requires less acid for its decalcification.

I cannot subscribe to the view held by some that no lime salts in a state of solution are to be found in the mass of decayed dentine. It may be easily shown that the amount of lime salt in solution which we could expect to find at any one time in a piece of carious dentine, as large, for instance, as half a pea, which has been a year in forming, would be so small as easily to escape detection. I have, however, with three or four such pieces, very seldom failed to find the salts in question. I make the test in the following manner: Three or four freshly extracted teeth, containing large quantities of softened dentine, are carefully washed to remove all traces of food. The softened dentine and enamel is then thoroughly removed, and placed in a small glass vessel with about four c. cm. of distilled water, (the larger pieces are cut into slices with a sharp, clean knife) and allowed to stand for half an hour. It is then filtered through three thicknesses of moistened Swedish filter-paper, the filtrate strongly acidulated with pure concentrated nitric acid, and any excess of the solution of molybdate of ammonium added, when it is briskly shaken and placed in an oven having a temperature of forty degrees Centigrade. Sometimes in a few hours, at other times in two to three days, yellow crystals of phospho-molybdate of ammonium will be deposited on the sides and bottom of the test tube. The solution of molybdate of ammonium is prepared as follows:

| | | |
|---|---|---|
| I. | Molybdate of Ammonium, | 100 grams. |
| | Ammonia, | 400 Cc. |
| | Distilled Water, | 600 Cc. |

II. { Pure Concentrated Nitric Acid, 1000 Cc.
{ Distilled Water, 500 Cc.

The first is poured into the second.

We should beware of the statement that free acid cannot exist in contact with tooth tissue. My experiment shows that it can and does exist in contact with sound enamel, at least four months, without perceptibly affecting it. This error arose no doubt from the assumption that acids act upon the inorganic constituents of a tooth in the same manner as upon a simple salt of lime. There is, however, a vast difference ; we have in tooth bone not a simple carbonate or phosphate of lime, but a definite chemical combination of these salts with the organic matter of the tooth.

If, therefore, from any cause the tooth is defectively constructed, imperfectly calcified, containing numerous interglobular spaces, soft and porous, or if through any general or special disorder of the system, or repeated pregnancy, a removal of the lime salts already deposited takes place, or any weakening in the bond of union between the lime salts and the organic matter, the tooth does not thereby become carious, but acquires a predisposition to caries.

Not only is it possible to produce, as above described, artificial caries which to the naked eye exactly resemble natural caries, but the microscopical changes may be equally well produced. Place a piece of dentine which has lain for three months in the normal mixture, in a test tube, cover with a drop of saliva and add enough bread to produce an acid reaction. Infect strongly with fungi from the human mouth, and allow it to remain for four weeks, being careful to keep the reaction acid by the addition of fresh saliva and bread if necessary, and then make microscopic preparations in the ordinary manner. Even preparations made from dentine which has, for some months, been exposed to the action of saliva and bread without the particular infection described above, show all the characteristics of natural caries.

I will demonstrate here a slide containing sections of dentine, some artificially, others naturally decayed. I leave it to any one who is able to decide which is which.

M. Schlencker, of St. Gallen, enumerates in his little work entitled

"*Ueber das Wesen der Zahnverderbniss,*" one hundred and thirteen different substances which repeatedly are brought in contact with the human teeth as food or medicine, nearly all of which are injurious to them, some producing effects visible to the naked eye in the space of five minutes.

I do not attach overweight to these agents, nor do I by any means ignore them ; a slight lesion of the enamel produced in this manner may constitute a point of beginning for the ordinary agents, and result in the production of caries in a place which otherwise would have remained intact, to say nothing of the ravages which are sometimes produced by such occasional agents alone. Schlencker relates a case in his own practice, where a patient with medium teeth had them totally ruined by following a grape cure.

We may well meet with cases of caries which we cannot account for, if we pass blindly over facts of this nature.

A school of investigators in America, who have received the name of "Bacterians," and who look upon *putrefactive* bacteria as a first, chief, and almost sole cause of caries, has been repeatedly directing attention to one result of their experiments, viz.: that the proportion of lime salts in carious dentine is nearly or quite the same as in normal dentine ; *i. e.* no decalcification has taken place.

Simple analyses of carious dentine, as well as of dentine softened in the mixture of bread and saliva, I published in the July number of the *Dental Cosmos*, and I need only repeat here that analyses of large pieces of carious dentine made by different persons, gave an average of twenty-six per cent of lime salts ; carious dentine just from the surface of the normal gave an average of fifty-seven and five-tenths per cent, and that the analyses of dentine decalcified in saliva and bread gave results identical with those obtained from analyses of carious dentine.

These analyses, however, I do not hold to be of much value in settling the question of the etiology of *caries dentium.* They simply show what, until recently, had so long been looked upon as a settled fact, and what must at once suggest itself to every man who has ever excavated a carious tooth, that a tolerably thorough decalcification of the dentine takes place in caries ; that the extent of the decalcification decreases as the distance below the surface increases (it could not well be otherwise); that on the border of the normal dentine the decalcification is comparatively slight, and that the same characteristics are presented by dentine softened in a mixture of saliva with various articles of food, as by carious dentine. We obtain no idea, however, as to how much the tissue of the tooth has lost, or how much the lime salts have lost; we do not even know from the analyses whether the organic matter has lost anything at all. To obtain a more satisfactory solution of this question I proceeded as follows: I procured three perfectly fresh teeth which contained large quantities of carious dentine. These teeth were washed in a gentle stream of water, to remove all remains of food, and the softened dentine removed, *in one piece*, with a spoon-shaped excavator. The joint volume of the pieces was then determined by an instrument specially constructed for the purpose, which gave the volume at once in cubic millimeters, and also by the ordinary picnometer. Then, (from the same teeth if possible) pieces of sound dentine were procured whose volume was determined in the same manner. The pieces were then dried for thirty hours at one hundred and five degrees Centigrade, and analysed. I give the result of one analysis.

| | | |
|---|---|---|
| 187.2 cubic millimeters of sound dentine weighed | – | 0.3600 |
| " " " " carious " " | – | 0.0821 |
| Loss | – | 0.2779 |

| | |
|---|---|
| The sound dentine gave on analysis 72.1 per ct. lime salts, | 0.2595 |
| " carious " " " " 26.3 " " | 0.0192 |
| Loss – | 0.2403 |

| | |
|---|---|
| The sound dentine contained 27.9 organic matter, – | 0.1004 |
| " carious " " 73.7 " " – – | 0.0605 |
| Loss – | 0.0399 |

The carious dentine had accordingly lost on the whole, seven-ninths of its original mass, the lime salts had lost twelve-thirteenths, and the organic matter two-fifths.

In plain words, the carious dentine had suffered an almost complete decalcification, only one-thirteenth of the original amount of lime salts being still present. The organic matter had suffered the comparatively small loss of two-fifths of its original amount. This loss is no doubt attributable, for the most part, to the direct action of the micro-organisms upon the more completely decalcified portions of the carious dentine.

The results of these experiments are too plain to require further explanation. They show an almost complete decalcification of the carious dentine, and a comparatively small reduction of the organic matter, also that the organic matter yields *last* to the destroying agents.

If there is any one who still believes that caries is due to an attack upon and destruction of the organic matter by fungi, and a consequent liberation of the lime salts, which are *subsequently* washed away by mechanical means,he will learn, farther on, more of the fallacies of such a belief.

From the experiments above described, and from the analyses of carious dentine, we can draw no other conclusion than that in the process of dental caries the destruction of the lime salts goes far in advance of the destruction of the organic constituents, nor can I see how any unprejudiced mind can contain any other idea than that the decalcification is the result of the action of some acid or acids. Of course we cannot be sure that there may not possibly be some agent of a neutral or alkaline reaction, which may be able in some way, or under some condition, to slightly decalcify the teeth. A question of much greater importance is this ; do the fungi found in the human mouth have any part in the production of the acid which effects the decalcification ?

I have already pointed out in the columns of the INDEPENDENT PRACTITIONER, that fungi are found in the human mouth which, beyond doubt have the power to produce acid fermentation, but although I have made a great many experiments and cultures for the purpose of determining, if possible, how many different species of fungi we have to deal with, and to what extent each is to be held responsible for the acid produced, yet the difficulties in the way of a permanent solution of the question are so numerous that I have not yet arrived at results which I would feel justified in presenting to you as conclusive. Having now, however, I venture to state, definitely established the important facts that the first stage of dental caries consists of a decalcification by acids, and the second stage of a destruction of the decalcified tissue by micro-organisms, I shall henceforth direct my whole attention to the question of the origin of the acids.

It would be a tiresome and, I hope, an unnecessary task for me to describe in detail the results which I have obtained from a microscopical examination of carious dentine, enamel and cement. This I have already done in the May and June numbers of the INDEPENDENT PRACTITIONER, in the January and July numbers of the *Dental Cosmos*, and at the Union Dental Convention, held at Springfield, Mass., in June last. I will, therefore, give but a short outline of the results. Primary caries of the enamel is almost, if indeed we may not say absolutely, independent of micro-organisms. Not only are they incapable of penetrating sound enamel, but even enamel whose integrity has severely suffered by the action of acids is generally free from any invasion. Caries of the enamel proceeding upon its inner surface, is likewise independent of organisms.

Whether the small amount of organic matter which remains after decalcification does or does not fall a prey to fungi, is a matter of no importance whatever. Its survival in the human mouth is, under all circumstances, an impossibility. No more can the fungi of tooth caries penetrate normal dentine. On the contrary, the normal dentine is, as a rule, separated from the infected dentine by a zone of softened, non-infected dentine, of considerable width ; nor does the advance of the fungi at all correspond in outline with the advance of the decalcification. In a word, the invasion of the fungi is always preceded by the decalcification of the dentine. Any one who has succeeded in overcoming the technical difficulties in the way of making perfect preparations of carious dentine, needs no other instrument than the naked eye to convince himself of the truth of this statement.

The dentine, having become thoroughly decalcified, owes its farther destruction to the action of fungi. No changes can be seen in the structure of the softened dentine when no fungi are present; where the fungi have invaded the tissue we find the tubules extended, often to many times their normal dimensions, with a varicose outline; farther on two or more tubules run together, through the destruction of the basis-substance. In this manner caverns originate in the dentine, filled with the remains of the decomposing tissue and micro-organisms. These are more frequent and larger the more we near the surface, and lead finally to the complete obliteration of the structure, or to the destruction of the tissue itself.

Primary Caries of the Cementum is very seldom met with, and its consideration need not occupy us long. The preparations which I have show a softening of the tissue, followed by the never-failing micro-organisms, which, however, do not penetrate the tissue to any appreciable depth. In some instances they may be found in the cement corpuscles, as well as in their offshoots. With a few exceptions, I have not been able to detect any evidences of an inflammatory action, such as occurs in bone corpuscles, and even in these instances I could not be certain that the change was due to an inflammatory process.

A few words must be given the inflammatory theory of decay. We notice in the first place a complete absence of all the cardinal symptoms of inflammation. Redness and heat of course we do not expect to find in a nonvascular organ. We might, however, expect some slight swelling. Especially is the perfectly painless character of the process, unless the pulp itself becomes affected, very remarkable, and it precludes the idea of any exudation, since the latter, in a tissue as resistant as dentine, ought by pressure upon the dentinal fibrils to give rise to the severest pain. It is also a significant fact that we utterly fail to produce caries of the teeth by any agent which might be employed to produce inflammation in any other part of the human body; wounds, contusions, fractures, partial or complete interruption of the nutrition, produce no symptoms of inflammation in a tooth. We may even crush the tooth with the forceps, destroy the pulp, partially cut off its external source of nourishment, (the pericementum) and there will still be no more indication of any inflammation or caries than when we began, and if by any mechanical contrivance we protect the remains of a tooth so violently treated from external agents, it will not become carious. Moreover, as long as pulpless teeth and human teeth inserted on pivots or plates decay as rapidly as living teeth, we are hardly justified, I think, in looking upon inflammation as a very important factor in the production of caries.

I have been still further convinced, by an examination of several hundreds of specimens, that after decalcification has taken place, the only change of any importance which occurs is produced by micro-organisms. There is no expansion of the tubules, no formation of caverns, no melting down of the tissue, no change whatever by which one could distinguish carious dentine from dentine artificially softened in some weak organic acid, where fungi are not present, nor have I succeeded in finding anything which I could look upon as analogous to any of the products of inflammation found in other parts of the human body. I have here a slide containing sections of carious dentine from living and from dead teeth, and sections of dentine in which the phenomena of caries were from beginning to end artificially produced, outside of the mouth. I hope that an opportunity will be given to any one who is desirous of making the attempt, to determine which is which.

If, then, acids perform so important a part in the production of caries, can we not prevent or reduce its ravages by alkaline mouth-washes? Probably not, and for reasons which are very evident. Take a piece of dentine which has become partially decalcified by lying two or three months in a mixture of bread and saliva, thoroughly remove every particle of bread from its surface, hold it for half an hour in a stream of water if you like, then dry the moisture from its surface, and press it against a piece of blue litmus paper to squeeze out some of the liquid in the tubules. The paper becomes instantly and intensely red. Exactly the same process goes on in a carious tooth, and the experiment may be repeated with carious dentine with equal success. The acid generated by fermentation is taken up into the softened dentine, or the saliva having penetrated the tubules there undergoes acid fermentation in the substance of the dentine itself. We might as well try to neutralize the pieces of dentine in a mixture of saliva and bread by pouring an alkaline solution on the outside of the vessel containing them, as to remove the acidity of a piece of dentine in a cavity, perhaps filled with food, by a superficial rinsing of the mouth with some weak alkaline mouth-wash.

The acid which has worked its way into the dentinal tubules, or has been generated there by fermentation, does not enter immediately into combination with the lime ; a certain time elapses before it passes through the tubules and comes in contact with the lime salts, and then to all appearance a certain time again elapses before decomposition takes place.

I may say that in my investigations into the cause of caries I have consumed, in the last two years, at the lowest estimate, eight thousand teeth, having had a daily supply of from five to twenty teeth.

Since last February, when I wrote the articles for the INDEPENDENT PRACTITIONER, I have made untiring experiments and investigations to determine the presence of acids in the human mouth, and their ability to produce the phenomena of the first stage of caries. I have with equal zeal sought for factors which might be taken into account in connection with acids, and while I have failed to find the latter, my faith in acids has increased constantly, until to-day I see the need of little or nothing more than organic acids and fungi to account for all the phenomena of dental caries.

Give me these two factors and I can produce caries which will deceive the most experienced operators and microscopists.

I may sum up in the following propositions the results of my investigations on the subject of *caries dentium*:

*First.* The contact of saliva with amylaceous or saccharine food, (not to speak of nitrogenous food,) or a solution of sugar or starch in saliva, kept at body temperature, invariably gives rise in four or five hours to a strong acid reaction, due to the generation of an organic acid.

*Second.* There must consequently be in the human mouth a constant though variable generation of acid, because of the impossibility of keeping the mouth perfectly free from food and from solutions of amyloids in saliva, which penetrate cracks, pits, and fissures, or are held by capillary attraction between the surfaces of teeth in contact, and there become acid by fermentation.

*Third.* The degree of acidity depends somewhat upon the length of time which has elapsed since partaking of food, and will be found greatest on rising in the morning.

*Fourth.* A cavity of decay in which saccharine or amylaceous food has remained for some hours, must and will be found, always and without exception, to have an acid reaction.

*Fifth.* The extent to which any tooth suffers from the action of the acid depends upon its density and structure, but more particularly upon the perfection of the enamel and the protection of the neck of the tooth by healthy gums. What we might call the perfect tooth would resist indefinitely the same acid to which a tooth of opposite character would succumb in a few weeks.

*Sixth.* An occasional possible absence of an acid reaction in a cavity of decay is no indication that acid has not participated in the production of the cavity. Little or no value can be attached to tests of the saliva alone.

*Seventh.* Any general or special disorder or condition of the system which results in the withdrawal of lime salts from a tooth, or in a lowering of its density, or in a weakening of the chemical union between the organic and inorganic matter of the tooth, renders it more liable to decay.

*Eight.* Strong acid and corroding substances brought but momentarily into the human mouth, may give rise to lesions of the enamel at points where the ordinary agents alone could never have begun.

*Ninth.* All the *macro*scopical appearances and characteristics of caries may be produced with the greatest exactness *out* of the mouth, simply by subjecting teeth to those acid mixtures which are constantly to be found *in* the mouth.

*Tenth.* The superficial layers of carious dentine undergo an almost, if not absolutely complete decalcification, which decreases as we approach the normal dentine. The same is true of dentine decalcified in saliva and bread.

*Eleventh.* The destruction of the organic constituents follows (not precedes) the decalcification, and is evidently for the most part to be ascribed to the action of fungi.

*Twelfth.* The fungi found in the human mouth do not participate *directly* in the process of decalcification. The exact part which they perform in the production of an acid reaction requires farther investigation.

*Thirteenth.* The fungi produce the most manifold anatomical changes in the softened dentine, resulting in the complete obliteration of the structure and final disappearance of the tissue in a mass of debris and fungi.

*Fourteenth.* The invasion of the micro-organisms is always preceded by the extraction of the lime salts.

*Fifteenth.* The destruction of the tissue remaining after decalcification, is effected almost wholly by fungi alone.

*Sixteenth.* Inflammation can hardly be looked upon as a very important factor in caries of the teeth.

*Seventeenth.* Caries of the enamel is purely chemical, the decalcification resulting at once in the complete dissolution of the tissue.

*Eighteenth.* Caries of cement runs a course analogous to caries of dentine, a softening of the tissue by acids, and following this its destruction by fungi; a slight inflammatory action on the part of the living matter in the corpuscles, is not to be excluded.

---

Right-handedness.—This extends very far along the animal series. Parrots hold their food by preference in the right foot, and, though we cannot speak positively, wasps, beetles and spiders seem to use the right anterior foot most commonly.

